# Comparative efficacy of balloon dilatation duration on patients with choledocholithiasis: a Bayesian network meta-analysis and systematic review

**DOI:** 10.1007/s00464-025-12168-4

**Published:** 2025-09-04

**Authors:** Liping Yang, Xinlei Zhang, Jinfang Yu, Zilan Qin, Ping Yue, Jinhui Tian, Yanxian Ren, Yanyan Lin, Wenbo Meng

**Affiliations:** 1 Department of General Surgery, The First Hospital of Lanzhou University, Lanzhou, Gansu China; 2 School of Nursing, Lanzhou University, Lanzhou, Gansu China; 3 The First Clinical Medical School of Lanzhou University, Lanzhou, 730000 Gansu China; 4 Evidence-Based Medicine Centre, Lanzhou University, Lanzhou, Gansu China; 5 Gansu Province Key Laboratory of Biological Therapy and Regenerative Medicine Transformation, Lanzhou, Gansu China

**Keywords:** Choledocholithiasis, Endoscopic papillary balloon dilatation, Sphincterotomy, Optimal dilatation time, Network meta-analysis

## Abstract

**Objective:**

To evaluate the application effects of different balloon dilation durations in endoscopic papillary balloon dilation (EPBD) and small endoscopic sphincterotomy with balloon dilation (ESBD) for biliary duct calculi by network meta-analysis and find the most appropriate dilation durations for both.

**Methods:**

PubMed, Web of Science, Cochrane Library, Embase, and other databases were searched for relevant randomized controlled trials (RCTs) published up to August 2024. Data were analyzed using R (V.4.2.3) and Stata (V.17.0).

**Results:**

A total of 12 RCTs were included, which involved 3284 patients and two treatment methods for common bile duct stones, namely ESBD and EPBD. There are ten balloon dilation durations in total. Among them, there are six balloon dilation durations (0 s, 5 s, 30 s, 60 s, 180 s, 300 s) in the ESBD group and four balloon dilation durations (20 s, 60 s, 180 s, 300 s) in the EPBD group. The network meta-analysis showed no statistical difference among different balloon dilation durations in the ESBD group. In EPBD, the 180 s group had a lower incidence of post-ERCP pancreatitis than the 20 s (OR = 0.00, 95% CI = 0.00–0.11), 60 s (OR = 0.00, 95% CI = 0.00–0.06), and 300 s (OR = 0.00, 95% CI = 0.00–0.29) groups. Regarding the success rate of stone removal in EPBD, compared with the 180 s group, the success rate of stone removal in the 20 s (OR = 0.00, 95% CI = 0.00–0.60), 60 s (OR = 0.00, 95% CI = 0.00–0.28), and 300 s (OR = 0.00, 95% CI = 0.00–0.20) groups was significantly lower. The results of the surface under the cumulative ranking curve showed that 180 s was the optimal balloon dilation duration, both in ESBD and EPBD.

**Conclusions:**

The results of network meta-analysis show that the optimal balloon dilation duration in ESBD and EPBD may be 180 s. Further direct comparison studies and longer-term follow-up are needed.

**Supplementary Information:**

The online version contains supplementary material available at 10.1007/s00464-025-12168-4.

Common bile duct (CBD) stones represent a prevalent issue across the world [1, 2]. Traditional surgery not only brings physical pain to patients but also increases the incidence of complications and prolongs the length of hospital stay. Endoscopic retrograde cholangiopancreatography (ERCP) has been promoted worldwide as a valuable diagnostic technique and—compared to traditional surgery—is considered a safer, easier, and more convenient alternative for dealing with bile duct stones and tumors [3]. Indeed, ERCP and endoscopic sphincterotomy (EST) have emerged as effective methods for removing CBD stones [4]. However, studies have reported that EST is a pathogenic factor for early postoperative complications in patients undergoing ERCP. At the same time, EST destroys the normal physiological structure of the Oddi sphincter, which can lead to permanent loss of papillary sphincter function and may potentially cause long-term complications such as recurrent cholangitis and incidence of bile duct stones [5, 6].

In recent years, with the rapid development of endoscopic surgery technology, endoscopic papillary balloon dilation (EPBD) is an alternative method that transiently relaxes the biliary sphincter, facilitating stone extraction while decreasing the chance of damage to the sphincter [7]. However, shorter balloon dilation times significantly increase the risk of post-ERCP pancreatitis (PEP) and long-term complications [8, 9]. Researchers have also explored EPBD after EST (EST + EPBD) or small endoscopic sphincterotomy with balloon dilation (ESBD) to treat CBD stones. Both of these are effective and safe endoscopic approaches for removing large or multiple CBD stones [10, 11]. Nevertheless, the reported duration of balloon dilation varies widely from 0 to 300 s [12–14]. Theoretically, shorter dilatation durations can reduce the operation and radiation times, but the advantages and disadvantages of different treatment measures and balloon dilatation durations are unclear, especially in PEP.

In this study, a Bayesian network meta-analysis and a systematic review were performed to evaluate the therapeutic effects of ten different balloon dilation times in EPBD and ESBD, to provide a scientific and reliable basis for clinical practice.

## Methods

### Study registration

This network meta-analysis was registered in the International Prospective Register of Systematic Reviews (PROSPERO: CRD42020221458) and was reported following the Preferred Reporting Items for Meta-Analyses (PRISMA) [15].

### Selection criteria

Studies meeting all of the following inclusion criteria were used: (1) Subject: patients with common bile duct stones treated with EPBD or ESBD; (2) Intervention: comparison of the time of balloon dilation for EPBD or ESBD. Times for balloon dilation included 0 s, 5 s, 20 s, 30 s, 60 s, 180 s, and 300 s; (3) Design: randomized controlled trial (RCT); (4) Outcomes: incidence rates of PEP and the success rate of stone removal.

We excluded studies meeting any of the following criteria: (1) incomplete information or repeated publication; (2) animal experiments; (3) studies not reporting the results.

Post-ERCP pancreatitis was defined as meeting 2 of the following 3 criteria: severe abdominal pain consistent with pancreatitis, serum amylase > 3 times the upper limit of normal within 24 h post-ERCP, or imaging evidence of pancreatitis on ultrasound or computed tomography [16]. PEP severity was stratified based on the Cotton consensus criteria [16].

Success was defined as complete stone removal (including mechanical lithotripsy), confirmed by absent filling defects on the final occlusion cholangiogram.

### Search strategy

We searched the following databases from inception to August 12, 2024, using a predefined search strategy (Supplement 1): PubMed, Web of Science, Cochrane Library, Embase, ClinicalTrials.gov, China National Knowledge Infrastructure Library (CNKI), Wanfang Data, and China Biomedical Literature Database (CBM). We checked reference lists of included studies for additional references. There was no restriction on the search language and the publishing language. Except for Chinese and English, all publications were translated by commercial translation services.

### Data extraction and quality assessment

The retrieved records were imported into Endnote 21 document management software, and two reviewers (Xinlei Zhang and Jinfang Yu) independently screened titles, abstracts, and, if potentially eligible, full texts for inclusion. Disagreement was resolved by a third reviewer (Zilan Qin). The extracted data mainly included the basic information of the study (first author, year of publication, and country), baseline characteristics of subjects (operation method, balloon dilatation duration, sample size, gender, average age, mean number of stones, maximum stone size and balloon catheter diameters), and outcomes.

The quality assessment of the selected trials was implemented by using the Cochrane risk-of-bias tool [17]. RCT evaluation includes the following seven domains: (1) random sequence generation, (2) allocation concealment, (3) blinding of participants and personnel, (4) blinding of outcome assessment, (5) incomplete outcome data, (6) selective reporting, and (7) other bias.

### Data synthesis and statistical analysis

Stata (V.17.0) software was used to construct the network diagram and to exclude the interventions that could not form the network diagram. The nodes of the network diagram represent different interventions and the lines between nodes represent direct comparisons between the connected interventions, while the size of the node or the thickness of the line represents the sample size of the intervention or the number of included analysis studies, respectively. Bayesian NMA was performed using the Markov Chain Monte Carlo (MCMC) method in the R (V.4.2.3) software with GeMTC (V.0.14.3) and JAGS (V.4.3.1) packages. We assessed dichotomous outcomes and estimated the treatment effect for each pairwise comparison using the OR and a 95% CI. Iteration convergence was judged by iteration trajectory, where the number of pre-iterations and iterations is set to 50,000 and 100,000, respectively. The MCMC chain reaches stable fusion from the initial part, and the convergence degree meets the requirements. Due to the characteristics of Bayesian statistical analysis, each result in the study is a closed loop, and the node-splitting analysis model is used to evaluate the degree of inconsistency. If the node-splitting analysis shows *P* < 0.05, it indicates that there is significant inconsistency. Then, compute the cumulative ranking probabilities using the surface under the cumulative ranking curves (SUCRA). A SUCRA value of 1.00 indicates a treatment certain to be the best; a value of 0, certain to be the worst. Publication bias was evaluated by visual inspection of funnel plot asymmetry if necessary.

## Results

### Identification of relevant studies

**Fig. 1:**
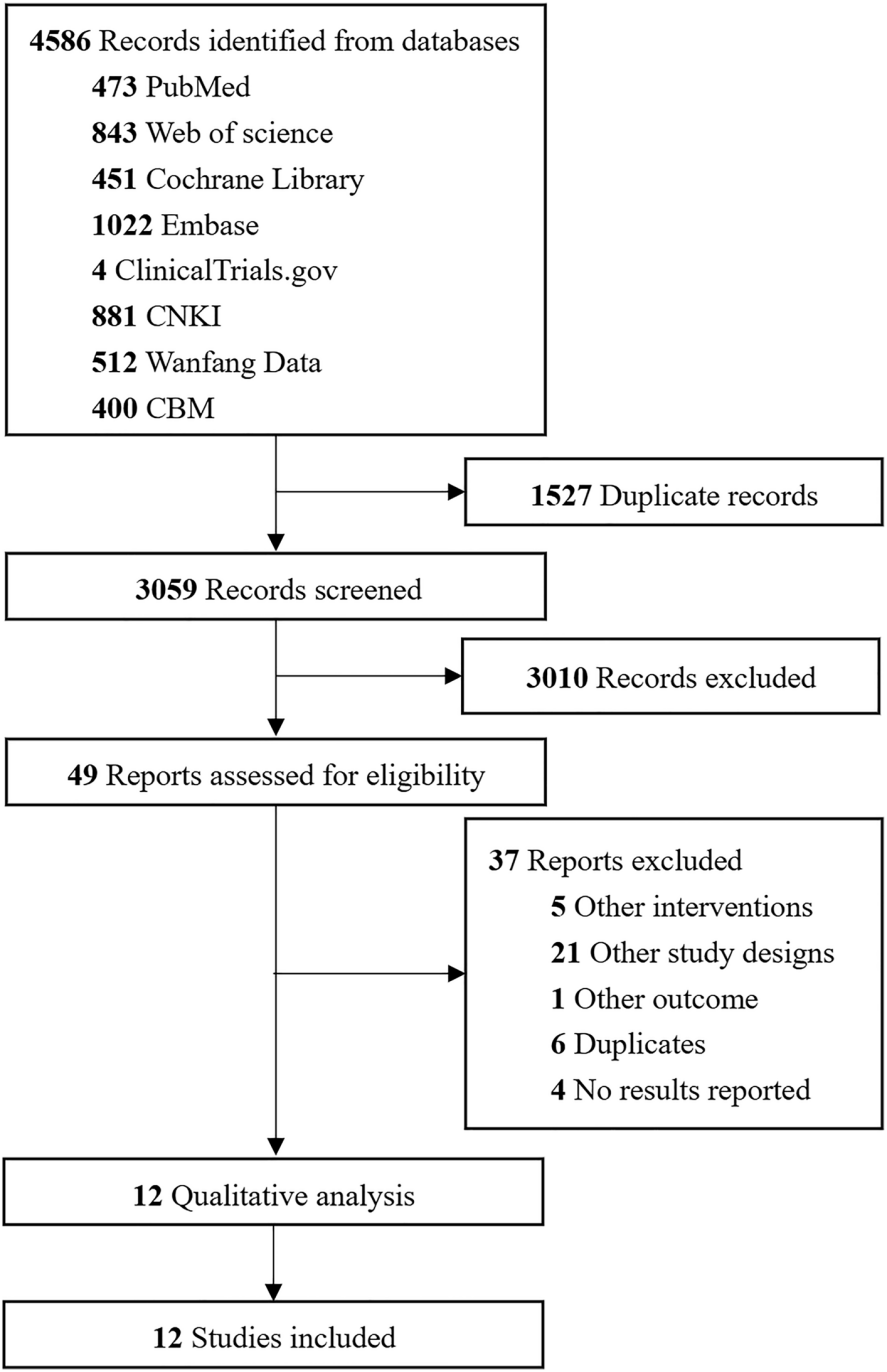
Flow chart of the literature searching and screening process

4586 related studies were retrieved through the pre-defined retrieval method. After processing in Endnote 21 software and manual removal of duplicate studies, 3059 studies remained. After primary screening of the article titles and abstracts, 3010 studies were excluded, and 37 additional studies were excluded after a secondary screening. Finally, 12 manuscripts were included for the present study. A total of 7 English and 5 Chinese articles were included, involving 10 balloon dilatation durations of ESBD and EPBD. The flow chart of literature screening is shown in Fig. 1.

### Characteristics of included studies and risk-of-bias assessment

**Fig. 2:**
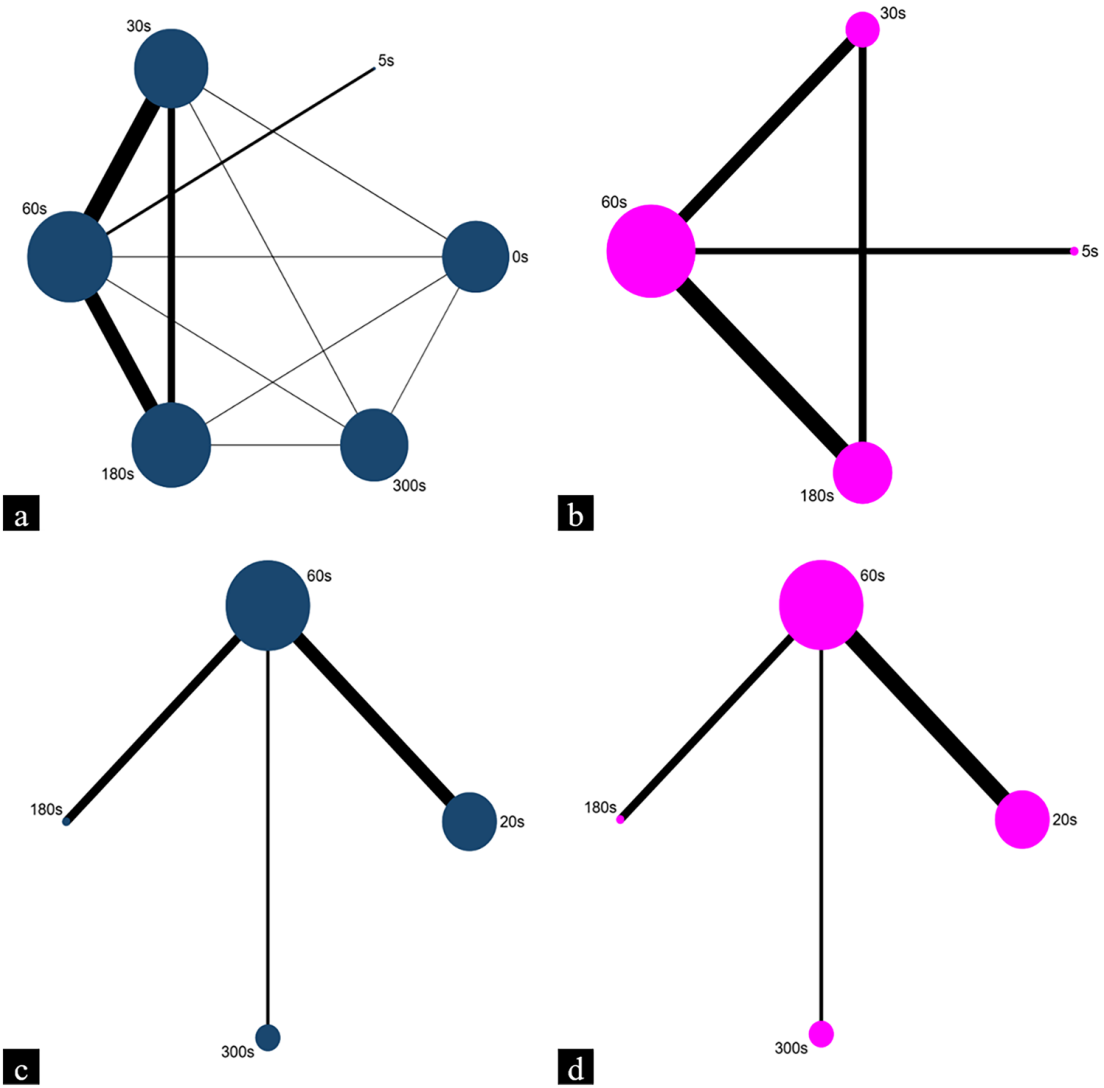
Networks for the duration of different balloon dilatation comparisons. **a** Post-ERCP pancreatitis in ESBD. **b** Successful stone removal in ESBD. **c** Post-ERCP pancreatitis in EPBD. **d** Successful stone removal in EPBD

Table 1 reports the characteristics of the included studies, which were published between 2010 and 2024 [12–14, 18–26]. Of the twelve studies, seven were in English [12–14, 18–20, 22], while five were in Chinese [21, 23–26]. Among them, ten were two-arm studies [13, 14, 18–23, 25, 26], while one was a three-arm study [24] and another one was a five-arm study [12]. Altogether, the twelve studies involved 3284 patients from four different countries, with eight studies from China [12, 13, 19, 21, 23–26], two from South Korea [18, 20], one from Greece [14], and one from Iran [22]. Two distinct treatment approaches were involved, including four EPBD [18–21] and eight ESBD [12–14, 22–26], with a total of ten different dilation times used. Network graphs for outcomes were plotted to show the comparative relationship between each intervention, with nodes representing the endoscopic approaches (Fig. 2).

**Table 1:** Characteristics of the included studies

References	Country	Operation	Intervention	Number of patients	Gender (male/female)	Age (years)	Number of stones	Maximum stone size (mm)	Balloon catheter diameters (mm)
Bang et al. [18]	Korea	EPBD	20 s	35	16/19	63.30 ± 13.60	‒	8.20 ± 3.30	9.60 ± 2.40
		EPBD	60 s	35	19/16	66.20 ± 17.40	‒	8.10 ± 3.50	9.70 ± 2.60
Liao et al. [19]	China	EPBD	60 s	86	41/45	64.70 ± 15.90	‒	6.00 (2.00–23.00)	‒
		EPBD	300 s	84	44/40	61.20 ± 17.40	‒	6.30 (2.00–30.00)	‒
Paspatis et al. [14]	Greece	ESBD	30 s	64	23/41	75.10 ± 11.90	2.14 ± 1.10	15.80 ± 4.30	15.08 ± 1.12
		ESBD	60 s	60	27/33	74.70 ± 13.20	2.15 ± 1.30	15.70 ± 5.20	15.22 ± 1.45
Bang et al. [20]	Korea	EPBD	20 s	109	58/51	62.00 ± 16.90	‒	6.50 ± 2.70	‒
		EPBD	60 s	119	74/45	63.70 ± 16.60	‒	6.90 ± 2.90	‒
Hao et al. [21]	China	EPBD	20 s	45	18/27	60.73 ± 12.06	‒	7.98 ± 1.55	9.73 ± 1.18
		EPBD	60 s	45	20/25	62.58 ± 12.40	‒	8.24 ± 1.23	9.56 ± 1.27
Shavakhi et al. [22]	Iran	ESBD	5 s	31	10/21	55.74 ± 20.70	1.13 ± 0.42	7.74 ± 2.96	12.22 ± 2.16
		ESBD	60 s	29	14/15	54.97 ± 19.83	1.38 ± 0.72	8.10 ± 3.65	12.22 ± 2.08
Dai et al. [23]	China	EPBD	60 s	22	10/12	48.00 ± 13.20	2.30 ± 1.20	13.00 ± 3.00	‒
		EPBD	180 s	23	12/11	45.00 ± 11.20	2.50 ± 1.30	12.00 ± 3.00	‒
Meng et al. [12]	China	ESBD	0 s	371	162/209	65.00(52.00–74.00)	‒	10.00 (8.00–12.00)	‒
		ESBD	30 s	384	181/203	64.00(52.00–76.00)	‒	10.00 (8.00–12.00)	‒
		ESBD	60 s	388	183/205	65.00(52.00–76.00)	‒	10.00 (8.00–12.00)	‒
		ESBD	180 s	390	191/199	64.00(55.00–75.00)	‒	10.00 (8.00–12.00)	‒
		ESBD	300 s	387	198/189	65.00(54.00–75.00)	‒	10.00 (7.00–12.00)	‒
Wang et al. [24]	China	ESBD	30 s	22	7/15	61.10 ± 17.00	‒	‒	11.10 ± 1.80
		ESBD	60 s	21	8/13	68.00 ± 10.60	‒	‒	11.20 ± 2.20
		ESBD	180 s	18	11/7	63.00 ± 15.20	‒	‒	11.70 ± 1.50
Li et al. [13]	China	ESBD	60 s	160	84/76	58.00(45.30–68.00)	‒	9.00 (8.50–10.00)	‒
		ESBD	180 s	160	87/73	61.50(50.00–72.00)	‒	9.00 (8.40–10.00)	‒
Liang et al. [25]	China	ESBD	60 s	58	32/26	69.27 ± 3.81	‒	12.25 ± 3.37	‒
		ESBD	180 s	58	35/23	68.62 ± 4.12	‒	12.73 ± 3.54	‒
Zhang et al. [26]	China	ESBD	30 s	40	26/14	68.70 ± 12.50	2.50 ± 1.80	10.30 ± 2.90	‒
		ESBD	60 s	40	28/12	70.10 ± 9.90	2.40 ± 1.80	9.20 ± 2.80	‒

Data are reported as numbers, mean ± SD, and median (range)

*EPBD* endoscopic papillary balloon dilation, *ESBD* small endoscopic sphincterotomy and balloon dilation, *s* second, *mm* millimeter

### Risk-of-bias assessment

**Fig. 3:**
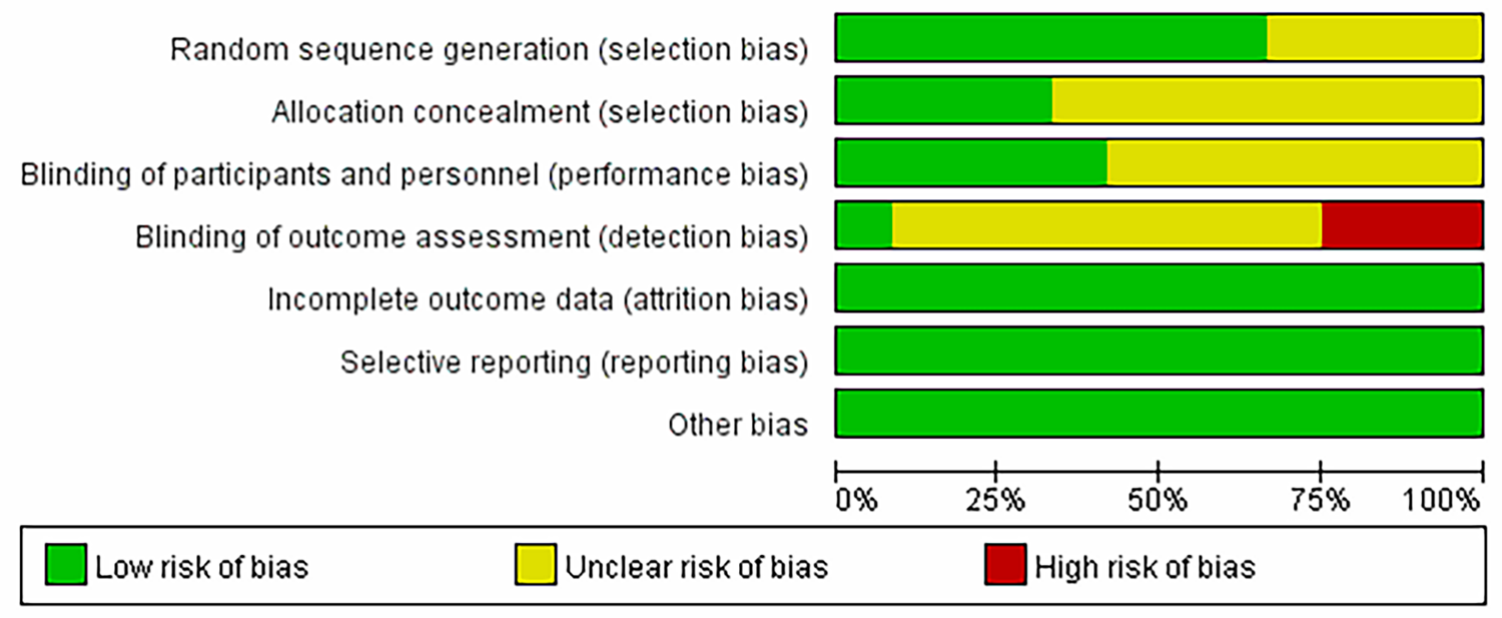
Results of bias assessed with the Cochrane risk-of-bias tool

Figure 3 shows the methodological quality assessment of the included studies. Eight studies reported the specific method of randomization—a random number table in all cases [12–14, 19, 22, 24–26]. Four studies reported allocation concealment [12–14, 19], and five studies reported blinding [12–14, 19, 22]. All data used were complete.

### Convergence test

As shown in the convergence diagnostic diagram (Appendix 1 in Supplement 2), for the consistency model analysis, all the PSRF values were 1.00 after 100,000 iterations, indicating that all the analyses have achieved good convergence.

### Node split

The node split models were constructed to assess inconsistencies between direct and indirect estimates in ESBD comparisons. In the groups of PEP and successful stone removal, all *P* values were greater than 0.05, demonstrating that no potential inconsistent risk existed. Regarding the comparison of different balloon dilation times in EPBD, a closed loop has not been formed, so there is no need to conduct an inconsistency test. Node split models are shown in Appendix 2 in Supplement 2.

### Direct comparison

In terms of the incidence of pancreatitis in ESBD, the meta-analysis found that compared with the 300 s group, the incidence of pancreatitis in the 30 s (OR = 0.44, 95% CI = 0.27–0.70), 60 s (OR = 0.50, 95% CI = 0.32–0.79), and 180 s (OR = 0.57, 95% CI = 0.36–0.88) groups was significantly lower; and the incidence of pancreatitis in the 0 s group was higher than that in the 30 s (OR = 1.71, 95% CI = 1.04–2.81). There was no statistically significant difference in the balloon dilation time of other outcome indicators between the two groups. The results of the meta-analysis are shown in Table 3a in Supplement 2.

In terms of the incidence of pancreatitis in EPBD, meta-analysis results indicated that the incidence of pancreatitis in the 60 s group was higher than that in the 300 s (OR = 3.56, 95% CI = 1.11–11.42). There was no statistically significant difference in the balloon dilation time of other outcome indicators between the two groups. The results of the meta-analysis are shown in Table 3b in Supplement 2.

### Bayesian network meta-analysis

#### Post-ERCP pancreatitis

According to network meta-analysis results, there was no statistical difference observed between the indirect comparison results of the incidence of PEP for different ESBD balloon dilatation durations (*P* > 0.05) (Table 4a in Supplement 2). Display the possible ranking of balloon dilation times by SUCRA. The ranking of ESBD pancreatitis incidence from the lowest (first place) to the highest (last place) is as follows: 180 s, 5 s, 30 s, 60 s, 0 s, 300 s (Table 5a in Supplement 2).

In EPBD, the incidence of PEP in the 180 s group was lower than that in the 20 s group (OR = 0.00, 95% CI = 0.00–0.11), the 60 s group (OR = 0.00, 95% CI = 0.00–0.06), and the 300 s group (OR = 0.00, 95% CI = 0.00–0.29) (Tables 4c in Supplement 2). The ranking of EPBD pancreatitis incidence from the lowest (first place) to the highest (last place) is as follows: 180 s, 300 s, 20 s, 60 s (Table 5b in Supplement 2).

#### Successful stone removal

According to network meta-analysis results, there was no statistical difference observed between the indirect comparison results of the incidence of successful stone removal for different ESBD balloon dilatation durations (*P* > 0.05) (Tables 4b in Supplement 2). The ranking of ESBD stone removal success from the highest (first place) to the lowest (last place) is as follows: 180 s, 30 s, 60 s, 5 s (Table 5a in Supplement 2).

Regarding the success rate of stone removal in EPBD, compared with the 180 s group, the success rate of stone removal in the 20 s (OR = 0.00, 95% CI = 0.00–0.60), 60 s (OR = 0.00, 95% CI = 0.00–0.28), and 300 s (OR = 0.00, 95% CI = 0.00–0.20) groups was significantly lower (Table 4d in Supplement 2). The ranking of EPBD stone removal success from the highest (first place) to the lowest (last place) is as follows: 180 s, 20 s, 60 s, 300 s (Table 5b in Supplement 2).

### Comparison-adjusted funnel plot

**Fig. 4:**
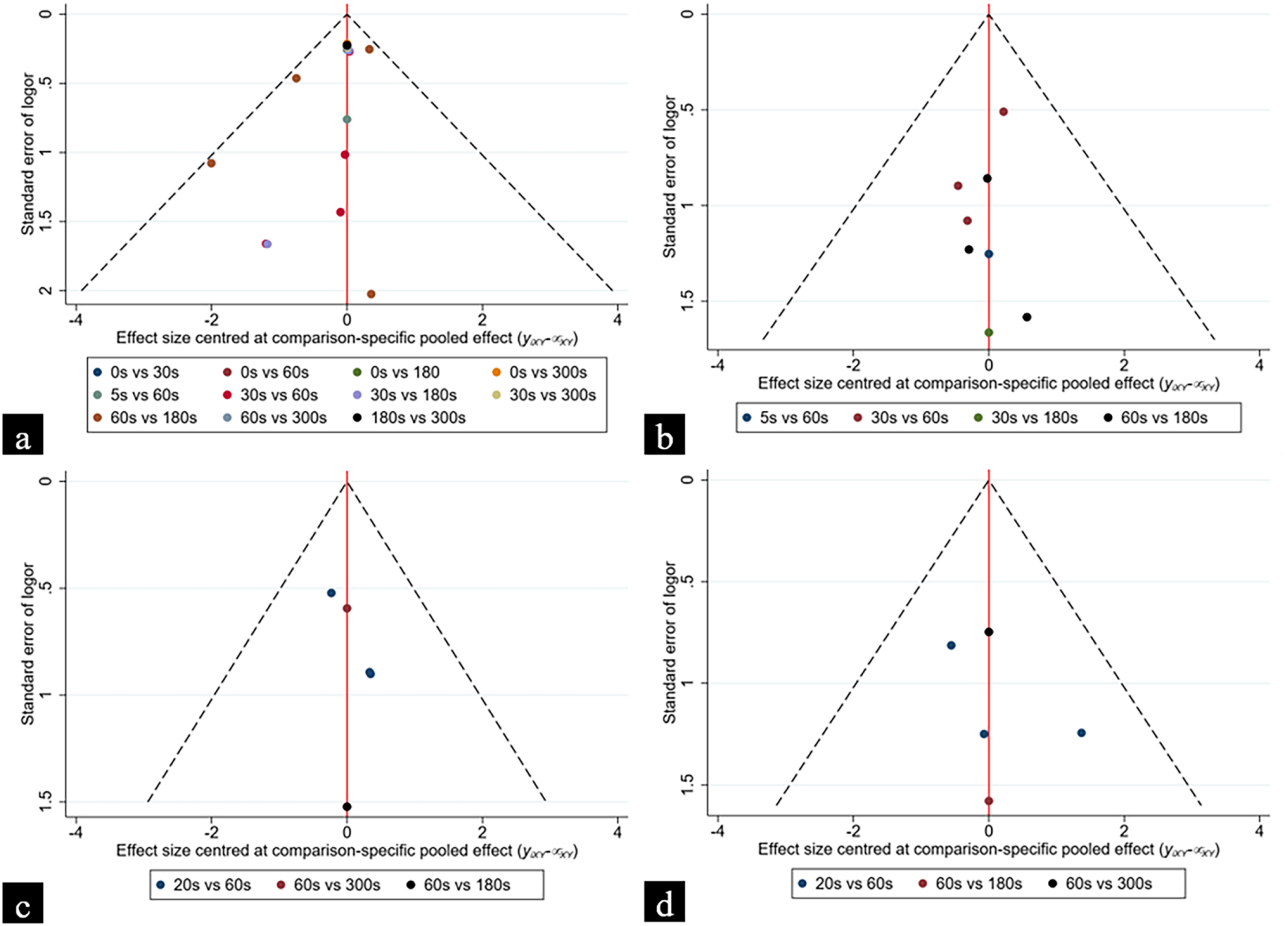
Funnel plots for the duration of different balloon dilatation comparisons. **a** Post-ERCP pancreatitis in ESBD. **b** Successful stone removal in ESBD. **c** Post-ERCP pancreatitis in EPBD. **d** Successful stone removal in EPBD

Based on the results of the four funnel plots (Fig. 4), each study is basically symmetrical on both sides of the funnel plot, so it can be considered that the current study has a relatively low probability of publication bias.

## Discussion

Pre-existing meta-analyses on the impact of balloon dilation durations in ESBD and EPBD on the incidence of PEP focused only on long- or short-term durations [27, 28]. This study is the first, to our knowledge, to perform a high-level meta-analysis comparing the impact of multiple balloon dilation times in ESBD and EPBD in patients with CBD stones. In the initial study, the direct comparison of balloon dilatation durations was different for ESBD and EPBD [12, 13, 19, 20]. Based on the existing research, network meta-analysis provides a method to indirectly compare the balloon dilation times for ESBD and EPBD. Based on this, the present study employed a network meta-analysis to combine direct and indirect comparison results and quantitatively ranked the balloon dilation times to obtain the optimal balloon dilation time, thus providing a basis for the early recovery of gallstone patients and reducing the occurrence of undesirable postoperative complications.

EPBD is an alternative method of endoscopic sphincterotomy [4]. Compared with EST, EPBD has a lower risk of bleeding and is thus more suitable for patients with coagulation dysfunction and stones that are difficult to remove [29, 30]. Recent research reports EPBD has sufficient efficacy in the treatment of CBD stones and is similar to traditional EST in that regard [31]. The most common complication of EPBD in the treatment of CBD stones is PEP [9]. Previous studies have shown that the balloon dilation time may be related to the incidence of PEP [19]. A network meta-analysis [32] shows that, compared with the balloon dilation time of less than one minute in EPBD, the incidence of pancreatitis is lower for the balloon dilation time of 2–5 min. A dilation time of 1 min or less is considered insufficient to relax the intact sphincter of Oddi, resulting in compartment syndrome and edema, which could increase the frequency of PEP [33, 34]. However, the current research has a relatively rough classification of the duration of balloon dilation, and the optimal balloon dilatation duration has not yet been determined.

In our study, we analyzed 12 RCTs, including 3284 patients and 2 operation methods, and evaluated the impact of 10 different balloon dilation times on the incidence of PEP and the successful stone removal rate. The results of the indirect comparison in this network meta-analysis showed that there were no significant differences in the incidence of PEP and the successful stone removal rate at the different balloon dilatation durations for ESBD. Notably, the incidence of PEP in this study for ESBD was slightly different from previous studies [12, 13]. It is speculated that this may be related to the unclear mechanism of PEP occurrence, which might be associated with the patient’s anatomical structure, mechanical lithotripsy, and repeated lithotomy. However, the SUCRA indicates that a balloon dilatation of 180 s may be a relatively optimal choice, as it tends to have a relatively low incidence of PEP and a relatively high successful stone removal rate. This is presumably because the small incision of the sphincter can effectively guide the balloon to expand in the direction of the bile duct sphincter, and appropriate balloon dilation time reduces the edema around the sphincter of the pancreas, which is conducive to the outflow of pancreatic juice and, thus, reduces the incidence of PEP [35]. Theoretically, a longer duration of balloon dilatation helps relax the Oddi sphincter and expose the surgical site, thus facilitating a more thorough removal of CBD stones. But prolonged compression on the pancreatic orifice by the inflated balloon could cause more edema and impaired drainage from the pancreas [12].

The systematic review by Liao et al. [7] showed that short-term expansion can increase the incidence of PEP, mainly due to the inability of a brief dilation to completely relax the Oddi sphincter and also partially due to the increased risk of fascial compartment syndrome and edema, which may further increase the incidence of PEP in EPBD [33, 34]. Among patients who receive EPBD, a dilation duration shorter than 3 min is an independent risk factor for PEP [36]. The guidelines issued by the European Society for Gastrointestinal Endoscopy recommend that the duration of EPBD should exceed 2 min. The results of this study also show that 180 s of balloon expansion is the optimum balloon dilatation duration in EPBD, exhibiting a trend of reducing the incidence rate of PEP. More effective, longer-term balloon dilatations in EPBD will reduce the number of operations for repeated stone removal and mechanical lithotripsy by completely relaxing the sphincter of Oddi and reducing the edema around the pancreatic duct [19].

The methodological quality of the 12 RCTs incorporated in this study was all at the intermediate-high level, and the sample sizes of these trials varied from 45 to 1920 participants. Our study has the following advantages: (1) By using a retrieval strategy that combines keywords and subject headings, papers from a wide range of fields were retrieved, which minimized the publication bias. (2) Only randomized controlled trials were selected. (3) Bayesian network meta-analysis was utilized to compare the balloon dilatation durations in EPBD and ESBD in terms of the incidence of PEP and the successful stone removal rate.

Yet, this study has some limitations, as follows: (1) Some of the included RCTs did not report the specific methods for generating random sequences, the application of blinding methods, and the situation of concealed allocation. (2) Due to the small amount of economic data retrieved, it was not possible to evaluate the economic cost of balloon dilation. (3) The follow-up duration in the retrieved literature was relatively short, with no records of late-stage complications. Consequently, it was infeasible to analyze late-stage complications. (4) The included studies did not sufficiently report PEP severity data, so the correlation between balloon dilation time and PEP severity could not be evaluated.

## Conclusion

Based on the results of this study, a balloon dilatation duration of 180 s in both ESBD and EPBD operations is the optimal choice, which can effectively reduce the occurrence of PEP and increase the success rate of stone removal. This is of great significance in future clinical practice and research. Of course, clinicians should fully understand the mechanisms of action and the desired function of balloon dilatation for ESBD or EPBD, be able to individually select the optimum balloon dilatation duration for their application, and then make individualized treatment plans for patients with CBD stones. Although there is insufficient evidence, more attention should be paid to conducting direct comparisons of balloon dilatation durations in this field.

## Supplementary Information

Below is the link to the electronic supplementary material.

Supplementary file1 (DOCX 19 KB)

Supplementary file2 (DOCX 230 KB)

## References

[1] Lammert F, Gurusamy K, Ko CW, Miquel JF, Méndez-Sánchez N, Portincasa P, van Erpecum KJ, van Laarhoven CJ, Wang DQ (2016) Gallstones. Nat Rev Dis Primers 2:16024 27121416 10.1038/nrdp.2016.24

[2] Shaffer EA (2006) Gallstone disease: epidemiology of gallbladder stone disease. Best Pract Res Clin Gastroenterol 20:981–996 17127183 10.1016/j.bpg.2006.05.004

[3] Neoptolemos JP, Carr-Locke DL, London NJ, Bailey IA, James D, Fossard DP (1988) Controlled trial of urgent endoscopic retrograde cholangiopancreatography and endoscopic sphincterotomy versus conservative treatment for acute pancreatitis due to gallstones. Lancet 2:979–983 2902491 10.1016/s0140-6736(88)90740-4

[4] Testoni PA, Mariani A, Aabakken L, Arvanitakis M, Bories E, Costamagna G, Devière J, Dinis-Ribeiro M, Dumonceau JM, Giovannini M, Gyokeres T, Hafner M, Halttunen J, Hassan C, Lopes L, Papanikolaou IS, Tham TC, Tringali A, van Hooft J, Williams EJ (2016) Papillary cannulation and sphincterotomy techniques at ERCP: European society of gastrointestinal endoscopy (ESGE) clinical guideline. Endoscopy 48:657–683 27299638 10.1055/s-0042-108641

[5] Krims PE, Cotton PB (1988) Papillotomy and functional disorders of the sphincter of Oddi. Endoscopy 20(Suppl 1):203–206 3049058 10.1055/s-2007-1018176

[6] Freeman ML, Nelson DB, Sherman S, Haber GB, Herman ME, Dorsher PJ, Moore JP, Fennerty MB, Ryan ME, Shaw MJ, Lande JD, Pheley AM (1996) Complications of endoscopic biliary sphincterotomy. N Engl J Med 335:909–918 8782497 10.1056/NEJM199609263351301

[7] Liao WC, Tu YK, Wu MS, Wang HP, Lin JT, Leung JW, Chien KL (2012) Balloon dilation with adequate duration is safer than sphincterotomy for extracting bile duct stones: a systematic review and meta-analyses. Clin Gastroenterol Hepatol 10:1101–1109 22642953 10.1016/j.cgh.2012.05.017

[8] Shen YH, Yang LQ, Yao YL, Wang L, Zhang YY, Cao J, He QB, Zou XP, Li YH (2017) Dilation time in endoscopic papillary balloon dilation for common bile duct stones. Surg Laparosc Endosc Percutan Tech 27:351–355 28737592 10.1097/SLE.0000000000000431 PMC5633320

[9] Fujisawa T, Kagawa K, Hisatomi K, Kubota K, Nakajima A, Matsuhashi N (2016) Is endoscopic papillary balloon dilatation really a risk factor for post-ERCP pancreatitis? World J Gastroenterol 22:5909–5916 27468185 10.3748/wjg.v22.i26.5909 PMC4948272

[10] Liu P, Lin H, Chen Y, Wu YS, Tang M, Lai L (2019) Comparison of endoscopic papillary large balloon dilation with and without a prior endoscopic sphincterotomy for the treatment of patients with large and/or multiple common bile duct stones: a systematic review and meta-analysis. Ther Clin Risk Manag 15:91–101 30666119 10.2147/TCRM.S182615 PMC6331186

[11] Hu J, Mu N, He Y (2022) Comparing the efficacy of endoscopic balloon dilation alone and combined with endoscopic sphincterotomy for common bile duct stone: a systematic review and meta-analysis. Ann Palliat Med 11:163–172 35144408 10.21037/apm-21-3557

[12] Meng W, Leung JW, Zhang K, Zhou W, Wang Z, Zhang L, Sun H, Xue P, Liu W, Wang Q, Zhang J, Wang X, Wang M, Shao Y, Cai K, Hou S, Li Q, Zhang L, Zhu K, Yue P, Wang H, Zhang M, Sun X, Yang Z, Tao J, Wen Z, Wang Q, Chen B, Shao Q, Zhao M, Zhang R, Jiang T, Liu K, Zhang L, Chen K, Zhu X, Zhang H, Miao L, Wang Z, Li J, Yan X, Wang F, Zhang L, Suzuki A, Tanaka K, Nur U, Weiderpass E, Li X (2019) Optimal dilation time for combined small endoscopic sphincterotomy and balloon dilation for common bile duct stones: a multicentre, single-blinded, randomised controlled trial. Lancet Gastroenterol Hepatol 4:425–434 31003961 10.1016/S2468-1253(19)30075-5

[13] Li YY, Miao YS, Wang CF, Yan J, Zhou XJ, Chen YX, Li GH, Zhu L (2024) Optimal dilation duration of 10 mm diameter balloons after limited endoscopic sphincterotomy for common bile duct stones: a randomized controlled trial. Sci Rep 14:971 38200057 10.1038/s41598-023-50949-w PMC10782008

[14] Paspatis GA, Konstantinidis K, Tribonias G, Voudoukis E, Tavernaraki A, Theodoropoulou A, Chainaki I, Manolaraki M, Chlouverakis G, Vardas E, Paraskeva K (2013) Sixty- versus thirty-seconds papillary balloon dilation after sphincterotomy for the treatment of large bile duct stones: a randomized controlled trial. Dig Liver Dis 45:301–304 23195665 10.1016/j.dld.2012.10.015

[15] Page MJ, McKenzie JE, Bossuyt PM, Boutron I, Hoffmann TC, Mulrow CD, Shamseer L, Tetzlaff JM, Akl EA, Brennan SE, Chou R, Glanville J, Grimshaw JM, Hróbjartsson A, Lalu MM, Li T, Loder EW, Mayo-Wilson E, McDonald S, McGuinness LA, Stewart LA, Thomas J, Tricco AC, Welch VA, Whiting P, Moher D (2021) The PRISMA 2020 statement: an updated guideline for reporting systematic reviews. J Clin Epidemiol 134:178–189 33789819 10.1016/j.jclinepi.2021.03.001

[16] Cotton PB, Lehman G, Vennes J, Geenen JE, Russell RC, Meyers WC, Liguory C, Nickl N (1991) Endoscopic sphincterotomy complications and their management: an attempt at consensus. Gastrointest Endosc 37:383–393 2070995 10.1016/s0016-5107(91)70740-2

[17] Sterne JAC, Savović J, Page MJ, Elbers RG, Blencowe NS, Boutron I, Cates CJ, Cheng HY, Corbett MS, Eldridge SM, Emberson JR, Hernán MA, Hopewell S, Hróbjartsson A, Junqueira DR, Jüni P, Kirkham JJ, Lasserson T, Li T, McAleenan A, Reeves BC, Shepperd S, Shrier I, Stewart LA, Tilling K, White IR, Whiting PF, Higgins JPT (2019) RoB 2: a revised tool for assessing risk of bias in randomised trials. BMJ 366:l4898 31462531 10.1136/bmj.l4898

[18] Bang BW, Jeong S, Lee DH, Lee JI, Lee JW, Kwon KS, Kim HG, Shin YW, Kim YS (2010) The ballooning time in endoscopic papillary balloon dilation for the treatment of bile duct stones. Korean J Intern Med 25:239–245 20830219 10.3904/kjim.2010.25.3.239 PMC2932935

[19] Liao WC, Lee CT, Chang CY, Leung JW, Chen JH, Tsai MC, Lin JT, Wu MS, Wang HP (2010) Randomized trial of 1-minute versus 5-minute endoscopic balloon dilation for extraction of bile duct stones. Gastrointest Endosc 72:1154–1162 20869710 10.1016/j.gie.2010.07.009

[20] Bang BW, Lee TH, Song TJ, Han JH, Choi HJ, Moon JH, Kwon CI, Jeong S (2015) Twenty-second versus sixty-second dilation duration in endoscopic papillary balloon dilation for the treatment of small common bile duct stones: a prospective randomized controlled multicenter trial. Clin Endosc 48:59–65 25674528 10.5946/ce.2015.48.1.59 PMC4323434

[21] Hao J, Xu M, Hao S, Zhang A, Yao J (2015) The effect of ballooning time on endoscopic papillary balloon dilatation for the bile duct stones(<10 mm). Chin J Endosc 21:703–705

[22] Shavakhi A, Minakari M, Ardestani MH, Sadeghizadeh A, Shavakhi S (2015) A comparative study of one minute versus five seconds endoscopic biliary balloon dilation after small sphincterotomy in choleducolithiasis. Adv Biomed Res 4:28 25709993 10.4103/2277-9175.150421 PMC4333434

[23] Dai W, Sun S, Ma G, Wang Q, Zhou J, Zhang J, Yang X (2016) Effectiveness and safety of different endoscopic papillary balloon dilation time in treatment of common bile duct stones. Chin J Endosc 22:35–38

[24] Wang X, Qu J, Yuan Z, Wang G, Pan R, Li K (2019) Effect of EST combined with different dilation duration of EPBD on the treatment of common bile duct stones. J Hepatopancreatobiliary Surg 31:536–539

[25] Liang G, Huang B, Yu S, Wang C, Jin Y (2024) Effects of different balloon dilation time on stone clearance rate,liver function,stress level and postoperative pancreatitis in elderly patients with bile duct stones undergoing EST. J Hebei Med Univ 45:172–177

[26] Zhang L, Xu C, Shan Y (2024) Randomized clinical trial of sEST combined with EPBD at different dilation times in the treatment of choledocholithiasis. Chin J Oper Procedures Gen Surg (Electronic Edition) 18:295–298

[27] Wang Q, Fu L, Wu T, Ding X (2021) The ballooning time in endoscopic papillary balloon dilation for removal of bile duct stones: a systematic review and meta-analysis. Medicine (Baltimore) 100:e24735 33725940 10.1097/MD.0000000000024735 PMC7982145

[28] Yu ZY, Liang C, Yang SY, Zhang X, Sun Y (2022) The therapeutic effect of balloon dilatation with different duration for biliary duct calculi: a network meta-analysis. J Minimal Access Surg 18:327–337 10.4103/jmas.JMAS_304_20 PMC9306115 35708376

[29] Zhao HC, He L, Zhou DC, Geng XP, Pan FM (2013) Meta-analysis comparison of endoscopic papillary balloon dilatation and endoscopic sphincteropapillotomy. World J Gastroenterol 19:3883–3891 23840129 10.3748/wjg.v19.i24.3883 PMC3699051

[30] Ye Q, Zhang J, Ou X, Zhou X, Zhu C, Li W, Yao J, Zhang G (2023) Efficacy and safety of three endoscopic techniques for small common bile duct stones (≤ 10 mm): a multicenter, retrospective, cohort study with propensity score matching. Surg Endosc 37:1863–1869 36253627 10.1007/s00464-022-09436-y

[31] Liu HD, Zhang Q, Xu WS, Jin S (2024) Clinical efficacy of laparoscopic cholecystectomy combined with endoscopic papillary balloon dilation in treatment of gallbladder stones with common bile duct stones: a retrospective study. World J Gastrointest Surg 16:1700–1708 38983353 10.4240/wjgs.v16.i6.1700 PMC11230032

[32] Yu ZY, Liang C, Yang SY, Zhang X, Sun Y (2022) The therapeutic effect of balloon dilatation with different duration for biliary duct calculi: a network meta-analysis. J Minimal Access Surg. 10.4103/jmas.JMAS_304_20 10.4103/jmas.JMAS_304_20 PMC9306115 35708376

[33] Kim TH, Kim JH, Seo DW, Lee DK, Reddy ND, Rerknimitr R, Ratanachu-Ek T, Khor CJ, Itoi T, Yasuda I, Isayama H, Lau JY, Wang HP, Chan HH, Hu B, Kozarek RA, Baron TH (2016) International consensus guidelines for endoscopic papillary large-balloon dilation. Gastrointest Endosc 83:37–47 26232360 10.1016/j.gie.2015.06.016

[34] Cheon YK, Lee TY, Kim SN, Shim CS (2017) Impact of endoscopic papillary large-balloon dilation on sphincter of Oddi function: a prospective randomized study. Gastrointest Endosc 85:782-790.e781 27597425 10.1016/j.gie.2016.08.031

[35] Ishii S, Fujisawa T, Ushio M, Takahashi S, Yamagata W, Takasaki Y, Suzuki A, Okawa Y, Ochiai K, Tomishima K, Kanazawa R, Saito H, Shiina S, Isayama H (2020) Evaluation of the safety and efficacy of minimal endoscopic sphincterotomy followed by papillary balloon dilation for the removal of common bile duct stones. Saudi J Gastroenterol 26:344–350 32719239 10.4103/sjg.SJG_162_20 PMC8019135

[36] Chou CK, Lee KC, Luo JC, Chen TS, Perng CL, Huang YH, Lin HC, Hou MC (2020) Endoscopic papillary balloon dilatation less than three minutes for biliary stone removal increases the risk of post-ERCP pancreatitis. PLoS ONE 15:e0233388 32453738 10.1371/journal.pone.0233388 PMC7250446

