# Supplementary material for: Comparative efficacy of balloon dilatation duration on patients with choledocholithiasis: a Bayesian network meta-analysis and systematic review
Source: Surg Endosc. 2025 Sep 4;39(10):6383–92. doi: 10.1007/s00464-025-12168-4 (PMC12500835; doi:10.1007/s00464-025-12168-4)
Supplement: Supplementary file 1 — Supplementary file1 (DOCX 19 KB) [file 464_2025_12168_MOESM1_ESM.docx]

**Specific search terms and search strategies for each database.**

**PubMed**

**1** (((Choledochal stones[Title/Abstract] OR gallstones[Title/Abstract] OR bile duct stones[Title/Abstract] OR Cholelithiasis[Title/Abstract] OR Choledocholithiasis[Title/Abstract] OR common bile duct[Title/Abstract]) OR ("Choledocholithiasis"[Mesh])) OR ("Gallstones"[Mesh])) OR ("Cholelithiasis"[Mesh])

**2** (endoscopic papillary balloon dilation[Title/Abstract] OR EPBD[Title/Abstract] OR endoscopic sphincter dilation[Title/Abstract] OR balloon dilatation[Title/Abstract] OR endoscopic sphincterotomy[Title/Abstract] OR EST[Title/Abstract] OR sEST[Title/Abstract] OR sphincterotomy[Title/Abstract] OR balloon angioplasty[Title/Abstract] OR balloon dilation[Title/Abstract] OR balloon inflation[Title/Abstract] OR endoscopic sphincterotomy with balloon dilation[Title/Abstract] OR ESBD[Title/Abstract] OR endoscopic biliary sphincterotomy[Title/Abstract] OR EBS[Title/Abstract] OR Sphincterotomy, Endoscopic[Title/Abstract] OR Sphincterotomy, Transduodenal[Title/Abstract]) OR ("Sphincterotomy"[Mesh] OR "Sphincterotomy, Endoscopic"[Mesh] OR "Sphincterotomy, Transduodenal"[Mesh])

**3** (((((("Randomized Controlled Trials as Topic"[Mesh] OR "Randomized Controlled Trial" [Publication Type] OR "Controlled Clinical Trials as Topic"[Mesh]) OR ("Clinical Trials as Topic"[Mesh])) OR ("Single-Blind Method"[Mesh] OR "Double-Blind Method"[Mesh])) OR ("Random Allocation"[Mesh])) OR ("Controlled Clinical Trial" [Publication Type])) OR ("Clinical Trial" [Publication Type])) OR (random*[Title/Abstract] OR clinical trial[Title/Abstract] OR RCT[Title/Abstract])

**4** 1 AND 2 AND 3

**Web of science**

**1** Choledochal stones OR gallstones OR bile duct stones OR Cholelithiasis OR Choledocholithiasis OR common bile duct

**2** endoscopic papillary balloon dilation OR EPBD OR endoscopic sphincter dilation] OR balloon dilatation OR endoscopic sphincterotomy OR EST OR sEST OR sphincterotomy OR balloon angioplasty OR balloon dilation OR balloon inflation OR endoscopic sphincterotomy with balloon dilation OR ESBD OR endoscopic biliary sphincterotomy OR EBS

**3** random*

**4** 1 AND 2 AND 3

**Cochrane Library**

**1** (Choledochal stones OR gallstones OR bile duct stones OR Cholelithiasis OR Choledocholithiasis OR common bile duct):ti,ab,kw

**2** MeSH descriptor: [Gallstones] explode all trees

**3** MeSH descriptor: [Cholelithiasis] explode all trees

**4** MeSH descriptor: [Choledocholithiasis] explode all trees

**5** 1 OR 2 OR 3 OR 4

**6** (endoscopic papillary balloon dilation OR EPBD OR endoscopic sphincter dilation OR balloon dilatation OR endoscopic sphincterotomy OR EST OR sEST OR sphincterotomy OR balloon angioplasty OR balloon dilation OR balloon inflation OR endoscopic sphincterotomy with balloon dilation OR ESBD OR endoscopic biliary sphincterotomy OR EBS OR Sphincterotomy, Endoscopic OR Sphincterotomy, Transduodenal):ti,ab,kw

**7** MeSH descriptor: [Sphincterotomy] explode all trees

**8** MeSH descriptor: [Sphincterotomy, Endoscopic] explode all trees

**9** MeSH descriptor: [Sphincterotomy, Transduodenal] explode all trees

**10** 6 OR 7 OR 8 OR 9

**11** ("randomized controlled trial" OR "controlled clinical trial" OR "clinical trial"):pt OR (random* OR "clinical trial"):ti,ab,kw

**12** MeSH descriptor: [Randomized Controlled Trials as Topic] explode all trees

**13** MeSH descriptor: [Clinical Trial] explode all trees

**14** MeSH descriptor: [Controlled Clinical Trials as Topic] explode all trees

**15** MeSH descriptor: [Random Allocation] explode all trees

**16** MeSH descriptor: [Double-Blind Method] explode all trees

**17** MeSH descriptor: [Single-Blind Method] explode all trees

**18** ("randomized controlled trial" OR "controlled clinical trial" OR "clinical trial"):pt

**19** (random* OR "clinical trial"):ti,ab,kw

**20** 11 OR 12 OR 13 OR 14 OR 15 OR 16 OR 17 OR 18 OR 19

**21** 5 AND 10 AND 20

**Embase**

**1** ('common bile duct stone'/exp OR 'common bile duct stone') AND [embase]/lim

**2** ('cholelithiasis'/exp OR 'cholelithiasis') AND [embase]/lim

**3** ('gallstone'/exp OR 'gallstone') AND [embase]/lim

**4** ('choledochal stones':ti,ab,kw OR gallstones:ti,ab,kw OR 'bile duct stones':ti,ab,kw OR cholelithiasis:ti,ab,kw OR choledocholithiasis:ti,ab,kw OR 'common bile duct':ti,ab,kw) AND [embase]/lim

**5** 1 OR 2 OR 3 OR 4

**6** ('endoscopic papillotomy'/exp OR 'endoscopic papillotomy') AND [embase]/lim

**7** ('endoscopic sphincterotomy'/exp OR 'endoscopic sphincterotomy') AND [embase]/lim

**8** ('sphincterotomy'/exp OR 'sphincterotomy') AND [embase]/lim

**9** ('endoscopic papillary balloon dilation':ti,ab,kw OR epbd:ti,ab,kw OR 'endoscopic sphincter dilation':ti,ab,kw OR 'balloon dilatation':ti,ab,kw OR 'endoscopic sphincterotomy':ti,ab,kw OR est:ti,ab,kw OR sest:ti,ab,kw OR sphincterotomy:ti,ab,kw OR 'balloon angioplasty':ti,ab,kw OR 'balloon dilation':ti,ab,kw OR 'balloon inflation':ti,ab,kw OR 'endoscopic sphincterotomy with balloon dilation':ti,ab,kw OR esbd:ti,ab,kw OR 'endoscopic biliary sphincterotomy':ti,ab,kw OR ebs:ti,ab,kw OR 'endoscopic papillotomy':ti,ab,kw) AND [embase]/lim

**10** 6 OR 7 OR 8 OR 9

**11** ('randomized controlled trial (topic)'/exp OR 'randomized controlled trial (topic)') AND [embase]/lim

**12** ('randomized controlled trial'/exp OR 'randomized controlled trial') AND [embase]/lim

**13** ('controlled clinical trial'/exp OR 'controlled clinical trial') AND [embase]/lim

**14** ('controlled clinical trial (topic)'/exp OR 'controlled clinical trial (topic)') AND [embase]/lim

**15** ('single blind procedure'/exp OR 'single blind procedure') AND [embase]/lim

**16** ('double blind procedure'/exp OR 'double blind procedure') AND [embase]/lim

**17** ('randomization'/exp OR 'randomization') AND [embase]/lim

**18** ('clinical trial'/exp OR 'clinical trial') AND [embase]/lim

**19** 'randomized controlled trial':it OR 'controlled clinical trial':it OR 'clinical trial':it

**20** ('clinical trial':ti,ab,kw OR random*:ti,ab,kw) AND [embase]/lim

**21** ('randomized controlled trial':ti,ab,kw OR 'controlled clinical trial':ti,ab,kw OR 'clinical trial':ti,ab,kw OR randomization:ti,ab,kw OR 'double blind procedure':ti,ab,kw OR 'single blind procedure':ti,ab,kw OR random*:ti,ab,kw) AND [embase]/lim

**22** 11 OR 12 OR 13 OR 14 OR 15 OR 16 OR 17 OR 18 OR 19 OR 20 OR 21

**23** 5 AND 10 AND 22

**CNKI**

**1** TKA%=胆总管结石 + 胆结石 + 胆管结石

**2** TKA%=EPBD + 十二指肠乳头球囊扩张术 + 内窥镜下十二指肠乳头球囊扩张术 + 乳头括约肌球囊扩张术 + 内窥镜下乳头括约肌球囊扩张术 + 乳头球囊扩张术 + 内镜下乳头球囊扩张术 + 内镜下球囊扩张术 + 球囊扩张术 + 气囊扩张术 + 内镜下气囊扩张术 + 内镜下括约肌扩张术 + 括约肌扩张术 + EST + sEST + ESBD + 内镜下十二指肠乳头括约肌切开术 + 十二指肠乳头括约肌切开术 + 内镜下乳头括约肌切开术 + 乳头括约肌切开术 + 内镜下括约肌切开术 + 括约肌切开术 + 内镜下乳头切开术 + 乳头切开术 + 乳头括约肌小切开术 + 内镜下乳头括约肌小切开术

**3** SU%=随机 OR FT%=随机 OR TKA%=随机

**4** 1 AND 2 AND 3

**Wang Fang Data**

**1** 主题:(胆总管结石 OR 胆石 OR 胆管结石 OR 胆结石)

**2** 主题:(EPBD OR 十二指肠乳头球囊扩张术 OR 内镜下十二指肠乳头球囊扩张术 OR 乳头括约肌球囊扩张术 OR 内镜下乳头括约肌球囊扩张术 OR 乳头球囊扩张术 OR 内镜下乳头球囊扩张术 OR 内镜下球囊扩张术 OR 球囊扩张术 OR 气囊扩张术 OR 内镜下气囊扩张术 OR 内镜下括约肌扩张术 OR 括约肌扩张术 OR EST OR sEST OR ESBD OR 内镜下十二指肠乳头括约肌切开术 OR 十二指肠乳头括约肌切开术 OR 内镜下乳头括约肌切开术 OR 乳头括约肌切开术 OR 内镜下括约肌切开术 OR 括约肌切开术 OR 内镜下乳头切开术 OR 乳头切开术 OR 乳头括约肌小切开术 OR 内镜下乳头括约肌小切开术)

**3** 全部:(随机)

**4** 1 AND 2 AND 3

**CBMdisc**

**1** "胆石症"[不加权:扩展] AND "胆石"[不加权:扩展] AND "胆总管结石病"[不加权:扩展]

**2** ("胆总管结石"[常用字段:智能] OR "胆石"[常用字段:智能] OR "胆管结石"[常用字段:智能] OR "胆总管结石病"[常用字段:智能] OR "胆结石"[常用字段:智能])

**3** "括约肌切开术, 内窥镜"[不加权:扩展]

**4** ("EPBD"[常用字段:智能] OR "十二指肠乳头球囊扩张术"[常用字段:智能] OR "内镜下十二指肠乳头球囊扩张术"[常用字段:智能] OR "乳头括约肌球囊扩张术"[常用字段:智能] OR "内镜下乳头括约肌球囊扩张术"[常用字段:智能] OR "乳头球囊扩张术"[常用字段:智能] OR "内镜下乳头球囊扩张术"[常用字段:智能] OR "内镜下球囊扩张术"[常用字段:智能] OR "球囊扩张术"[常用字段:智能] OR "气囊扩张术"[常用字段:智能] OR "内镜下气囊扩张术"[常用字段:智能] OR "内镜下括约肌扩张术"[常用字段:智能] OR "括约肌扩张术"[常用字段:智能] OR "EST"[常用字段:智能] OR "sEST"[常用字段:智能] OR "ESBD"[常用字段:智能] OR "内镜下十二指肠乳头括约肌切开术"[常用字段:智能] OR "十二指肠乳头括约肌切开术"[常用字段:智能] OR "内镜下乳头括约肌切开术"[常用字段:智能] OR "乳头括约肌切开术"[常用字段:智能] OR "内镜下括约肌切开术"[常用字段:智能] OR "括约肌切开术"[常用字段:智能] OR "内镜下乳头切开术"[常用字段:智能] OR "乳头切开术"[常用字段:智能] OR "乳头括约肌小切开术"[常用字段:智能] OR "内镜下乳头括约肌小切开术"[常用字段:智能] OR "括约肌切开术, 内窥镜"[常用字段:智能])

**5** "随机对照试验"[不加权:扩展]

**6** ("随机对照试验"[全部字段:智能] OR "随机对照试验研究"[全部字段:智能] OR "随机"[全部字段:智能] OR "RCT"[全部字段:智能])

**7** 1 OR 2

**8** 3 OR 4

**9** 5 OR 6

**10** 7 AND 8 AND 9
