# Supplementary material for: Comparative efficacy of balloon dilatation duration on patients with choledocholithiasis: a Bayesian network meta-analysis and systematic review
Source: Surg Endosc. 2025 Sep 4;39(10):6383–92. doi: 10.1007/s00464-025-12168-4 (PMC12500835; doi:10.1007/s00464-025-12168-4)
Supplement: Supplementary file 2 — Supplementary file2 (DOCX 230 KB) [file 464_2025_12168_MOESM2_ESM.docx]

Appendix 1 The convergence diagnostic diagram.


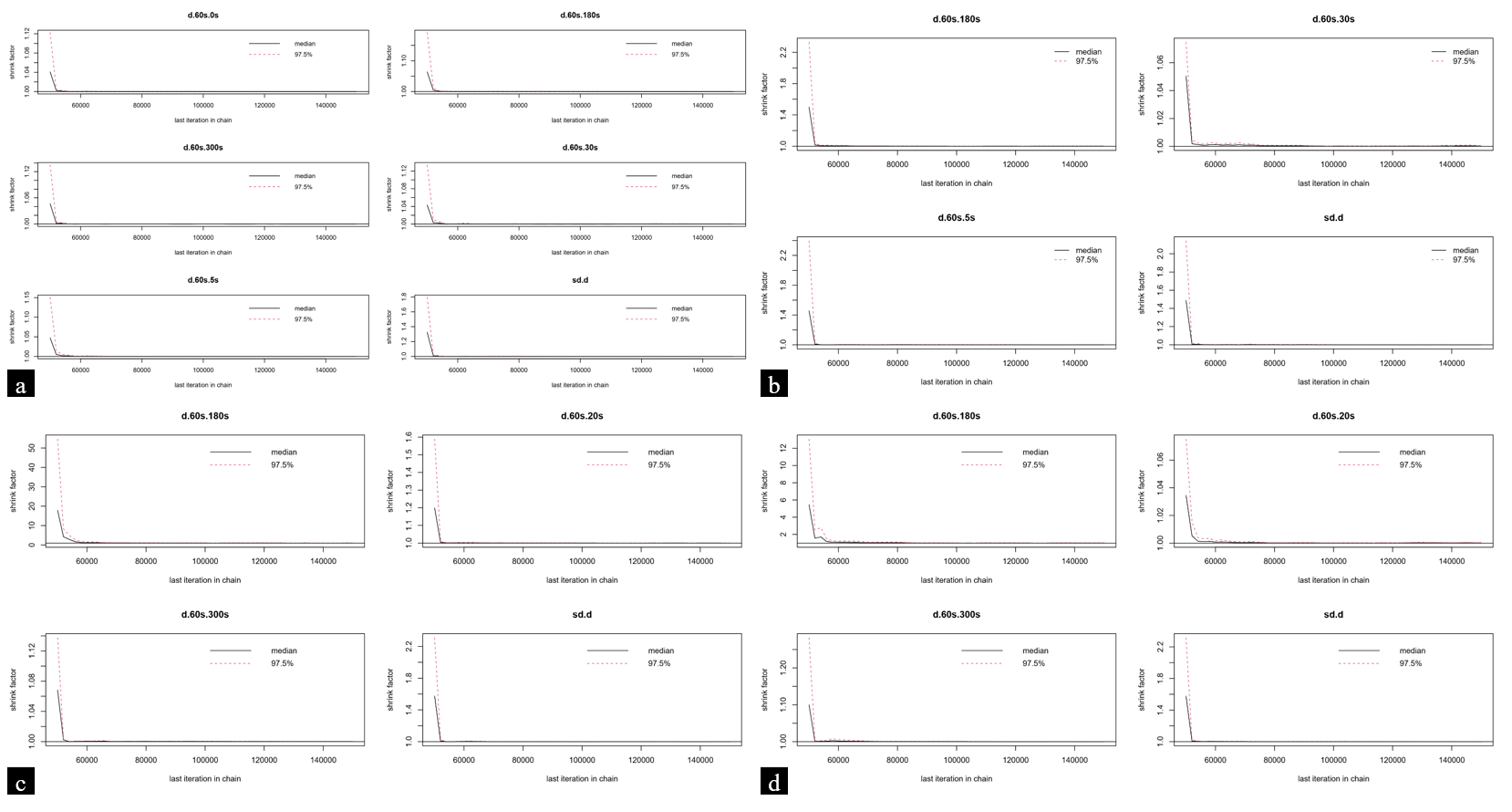


Figure 1 The convergence diagnostic diagram

(a) Post-ERCP pancreatitis in ESBD (b) Successful stone removal in ESBD

(c) Post-ERCP pancreatitis in EPBD (d) Successful stone removal in EPBD

Appendix 2 Node split models.


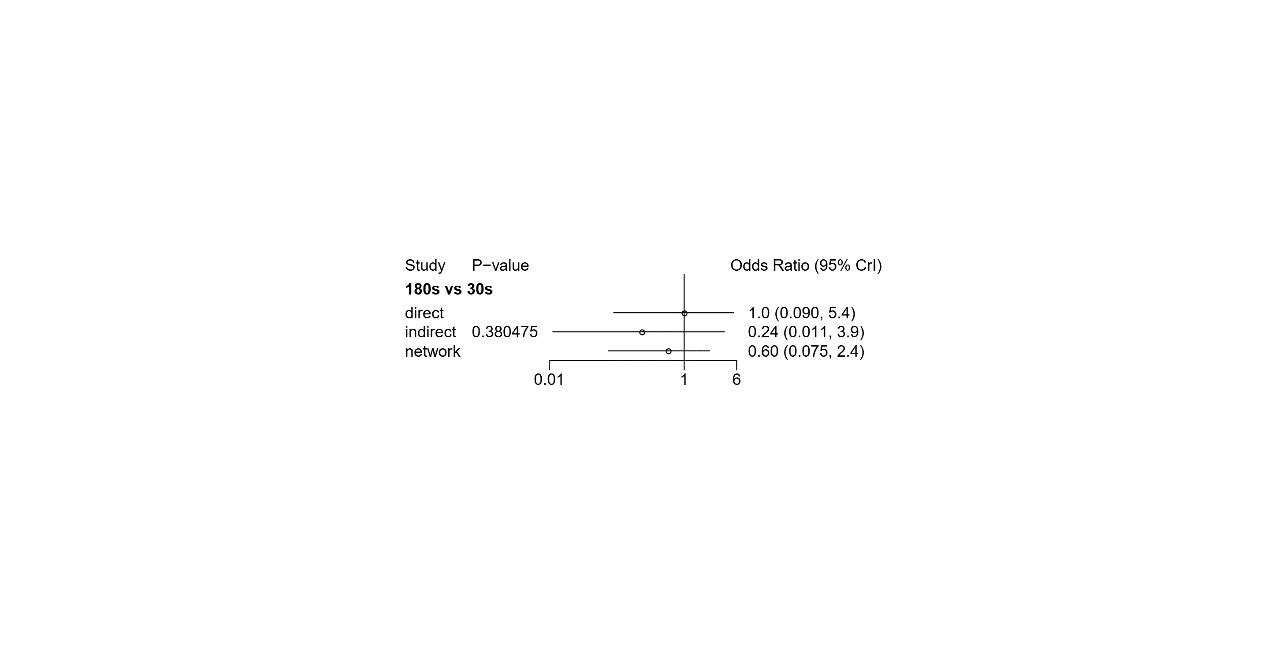


Figure 2a Post-ERCP pancreatitis in ESBD


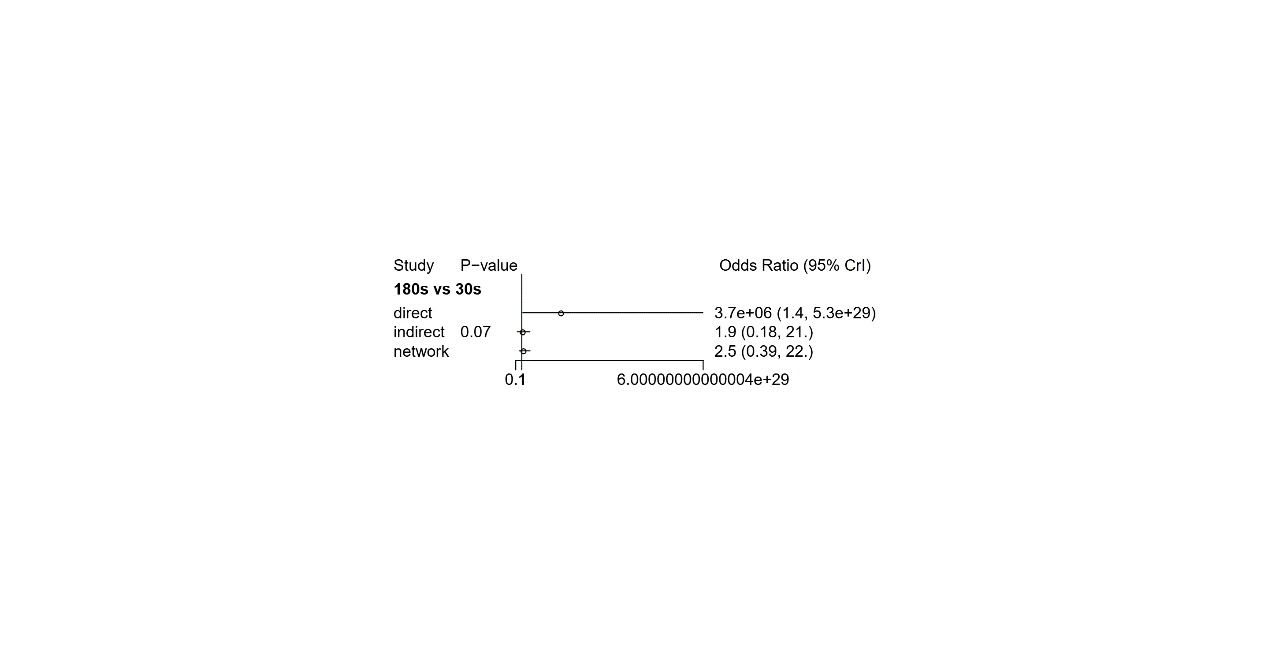


Figure 2b Successful stone removal in ESBD

Appendix 3 Direct comparison.

Table 3a Meta-analysis of different balloon dilatation time in ESBD

|  | post-ERCP pancreatitis | | successful stone removal | |
| --- | --- | --- | --- | --- |
| Interventions | Number of included studies | *OR (95%CI)* | Number of included studies | *OR (95%CI)* |
| 0s vs. 30s | 1 | 1.71 (1.04, 2.81) | - | - |
| 0s vs. 60s | 1 | 1.50 (0.93, 2.41) | - | - |
| 0s vs180s | 1 | 1.32 (0.83, 2.11) | - | - |
| 0s vs. 300s | 1 | 0.75 (0.49, 1.14) | - | - |
| 5s vs. 60s | 1 | 0.41 (0.09, 1.83) | 1 | 0.52 (0.04, 6.04) |
| 30s vs. 60s | 4 | 0.91 (0.56, 1.50) | 3 | 1.36 (0.61, 3.06) |
| 30s vs. 180s | 2 | 0.80 (0.48, 1.33) | 1 | 0.39 (0.02, 10.10) |
| 30s vs. 300s | 1 | 0.44 (0.27, 0.70) | - | - |
| 60s vs. 180s | 4 | 2.02 (0.65, 6.28)* | 2 | 0.33 (0.08, 1.41) |
| 60s vs. 300s | 1 | 0.50 (0.32, 0.79) | - | - |
| 180s vs. 300s | 1 | 0.57 (0.36, 0.88) | - | - |

* is the random-effects model, and the rest are fixed-effects models.

Table 3b Meta-analysis of different balloon dilatation time in EPBD

| Interventions | Number of included studies | post-ERCP pancreatitis | successful stone removal |
| --- | --- | --- | --- |
|  |  | *OR (95%CI)* | *OR (95%CI)* |
| 20s vs. 60s | 3 | 0.66 (0.30, 1.45) | 1.95 (0.65, 5.81) |
| 60s vs. 180s | 1 | 11.43 (0.58, 226.11) | 0.17 (0.01, 3.85) |
| 60s vs. 300s | 1 | 3.56 (1.11, 11.42) | 1.75 (0.41, 7.57) |

Appendix 4 Bayesian network meta-analysis.

Table 4a Post-ERCP pancreatitis in ESBD

| 0s | 0.34  (0.01, 8.39) | 0.73  (0.11, 6.78) | 0.88  (0.13, 6.22) | 0.46  (0.05, 2.77) | 1.34  (0.15, 11.99) |
| --- | --- | --- | --- | --- | --- |
| 2.97  (0.12, 79.79) | 5s | 2.22  (0.13, 52.80) | 2.62  (0.20, 38.64) | 1.31  (0.06, 22.72) | 3.98  (0.16, 105.80) |
| 1.37  (0.15, 8.79) | 0.45  (0.02, 7.59) | 30s | 1.19  (0.24, 4.42) | 0.61  (0.08, 2.41) | 1.84  (0.20, 11.56) |
| 1.14  (0.16, 7.88) | 0.38  (0.03, 5.01) | 0.84  (0.23, 4.11) | 60s | 0.51  (0.11, 1.58) | 1.52  (0.22, 10.37) |
| 2.19  (0.36, 19.85) | 0.76  (0.04, 16.03) | 1.65  (0.41, 12.69) | 1.97  (0.63, 9.00) | 180s | 2.92  (0.48, 26.69) |
| 0.75  (0.08, 6.69) | 0.25  (0.01, 6.33) | 0.54  (0.09, 5.03) | 0.66  (0.10, 4.64) | 0.34  (0.04, 2.09) | 300s |

Table 4b Successful stone removal in ESBD

| 5s | 3.29  (0.13, 173.50) | 2.34  (0.11, 98.54) | 8.86  (0.28, 569.10) |
| --- | --- | --- | --- |
| 0.30  (0.01, 7.87) | 30s | 0.72  (0.19, 2.34) | 2.58  (0.37, 23.61) |
| 0.43  (0.01, 8.78) | 1.39  (0.43, 5.14) | 60s | 3.58  (0.79, 24.71) |
| 0.11  (0, 3.51) | 0.39  (0.04, 2.69) | 0.28  (0.04, 1.27) | 180s |

Table 4c Post-ERCP pancreatitis in EPBD

| 20s | 1.64  (0.40, 8.07) | 0.00  (0.00, 0.11) | 0.43  (0.03, 8.12) |
| --- | --- | --- | --- |
| 0.61  (0.12, 2.50) | 60s | 0.00  (0.00, 0.06) | 0.26  (0.02, 2.92) |
| 4.594e+11  (9.12, 4.228e+36) | 7.65e+11  (16.27, 6.843e+36) | 180s | 1.983e+11  (3.48, 1.85e+36) |
| 2.33  (0.12, 39.46) | 3.81  (0.34, 46.48) | 0.00  (0.00, 0.29) | 300s |

Table 4d Successful stone removal in EPBD

| 20s | 0.53  (0.10, 2.87) | 3.029e+07  (1.67, 2.71e+25) | 0.28  (0.01, 5.80) |
| --- | --- | --- | --- |
| 1.89  (0.35, 9.90) | 60s | 5.738e+07  (3.55, 4.976e+25) | 0.54  (0.04, 6.44) |
| 0.00  (0.00, 0.60) | 0.00  (0.00, 0.28) | 180s | 0.00  (0.00, 0.20) |
| 3.53  (0.17, 71.48) | 1.84  (0.16, 23.98) | 1.105e+08  (4.94, 1.017e+26) | 300s |

Appendix 5 The results of the surface under the cumulative ranking curves (SUCRA).

Table 5a The results of the surface under the cumulative ranking curve in ESBD

| Interventions | post-ERCP pancreatitis | | successful stone removal | |
| --- | --- | --- | --- | --- |
|  | SUCRA | Rank | SUCRA | Rank |
| 0s | 0.36 | 5 | - | - |
| 5s | 0.74 | 2 | 0.20 | 4 |
| 30s | 0.50 | 3 | 0.56 | 2 |
| 60s | 0.40 | 4 | 0.35 | 3 |
| 180s | 0.77 | 1 | 0.89 | 1 |
| 300s | 0.23 | 6 | - | - |

Table 5b The results of the surface under the cumulative ranking curve in EPBD

| Interventions | post-ERCP pancreatitis | | successful stone removal | |
| --- | --- | --- | --- | --- |
|  | SUCRA | Rank | SUCRA | Rank |
| 20s | 0.34 | 3 | 0.54 | 2 |
| 60s | 0.10 | 4 | 0.31 | 3 |
| 180s | 0.99 | 1 | 0.99 | 1 |
| 300s | 0.57 | 2 | 0.16 | 4 |
